# Guide wire loss during central venous cannulation

**DOI:** 10.4103/0019-5049.72665

**Published:** 2010

**Authors:** Ashoo Wadehra, Pragati Ganjoo, Monica S Tandon

**Affiliations:** Department of Anaesthesiology and Critical Care, GB Pant Hospital, New Delhi, India

**Figure 1:**
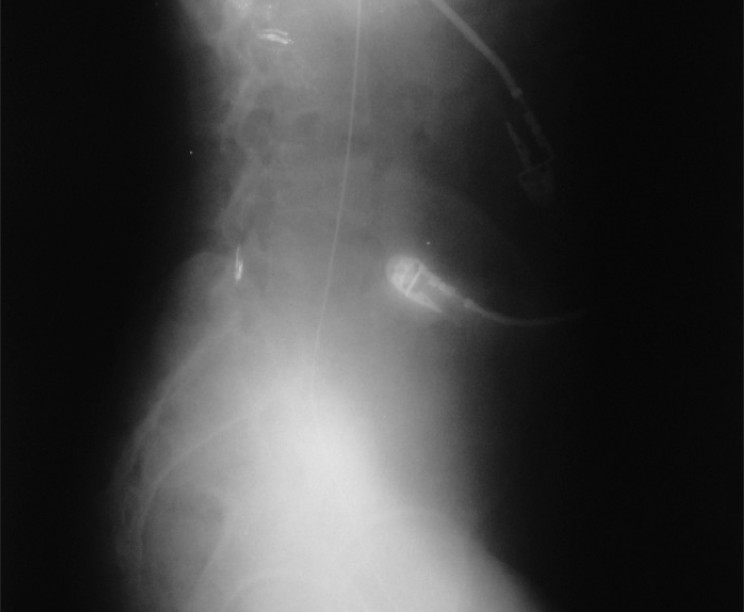
Migrated guide wire ascending in the inferior vena cava

**Figure 2:**
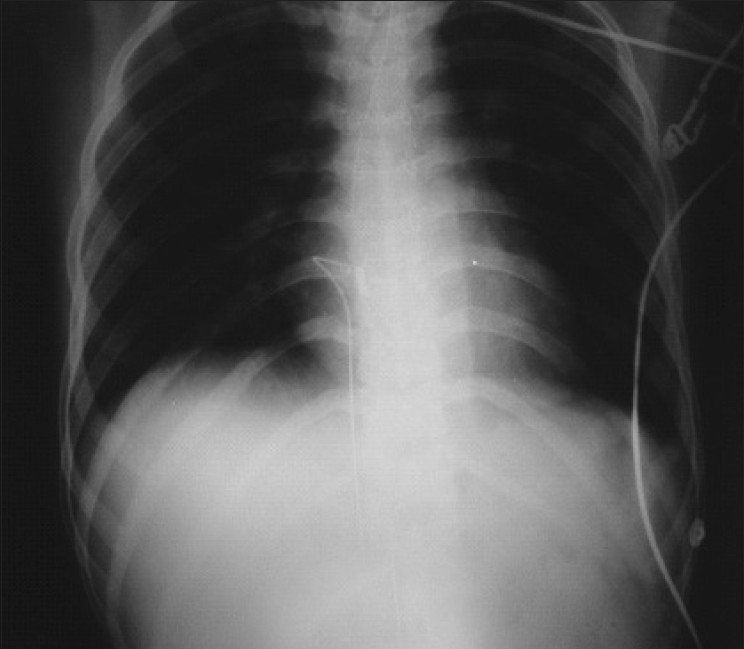
Intracardiac migration of the lost guide wire

Sir,

Central venous (CV) catheterisation is a routine procedure in intensive care units (ICU), with an overall complication rate of 12%. Loss of a complete guide wire into the circulation is one of its rare and preventable complications.[1] A 65-year-old male patient in our neurosurgical ICU required CV access on the second postoperative day, following an intracranial tuberculoma excision. CV catheterisation of the right femoral vein with the Seldinger technique was attempted in him by a first year resident doctor without supervision. During cannulation, an inadvertent leg movement by the drowsy patient caused the guide wire to slip out of the resident’s fingers, into the circulation. On radiography, the guide wire was seen to have ascended in the inferior vena cava and entered the heart [Figures 1 and 2]. The migrated wire did not produce any cardiac manifestations. Heparinisation of the patient for guide wire extraction was deferred for 2 days due to the recent neurosurgery. The guide wire was then removed percutaneously using a gooseneck snare under fluoroscopic guidance in the cardiac catheterisation laboratory.

Migration of a guide wire into the circulation can occur from any of the usual CV catheter insertion sites.[1–3] A complete guide wire may not necessarily produce any symptoms and its loss may remain unnoticed for long.[2] However, intravascular migration of a broken guide wire has the potential of causing adverse effects like vascular damage, thrombosis, embolism and arrhythmias; embolism from guide wire fragments can be fatal in up to 20% instances.[1] Cardiac tamponade manifesting 3 years after a guide wire loss has been reported as a late complication, highlighting the importance of wire extraction as soon as a diagnosis is made.[3] Retrieval is usually done by interventional radiology using gooseneck snares, endovascular retrieval forceps or Dormia baskets; surgical removal is also reported.[4]

The usual attributes for a guide wire loss are operator inexperience, inattention and inadequate supervision during catheterisation.[1] In our patient too, these were the main causes, compounded by the sudden movement of our disoriented patient. Expert operator skills and compliance with the catheterisation protocol are mandatory to prevent this complication. Firmly holding on to the tip of the guide wire at all times during catheterisation is the mainstay of prevention.[1] Prior sedation of disoriented patients may help achieve a smoother cannulation though sedatives need to be used cautiously in neurosurgical patients.
